# A EVOLUÇÃO DO HOSPITAL E A GESTÃO DA SAÚDE: ENTRE O LOCAL E O GLOBAL

**DOI:** 10.1590/S0104-59702024000100050

**Published:** 2024-10-14

**Authors:** Isabel Amaral, Alexandra Esteves, Céline Paillette

**Affiliations:** i Editora convidada, Centro Interuniversitário de História das Ciências e da Tecnologia/NOVA Faculdade de Ciências e Tecnologia. Lisboa – Portugal; ii Editora convidada, Universidade do Minho. Braga – Portugal; iii Editora convidada, Université Paris 1-Panthéon Sorbonne. Paris – France

A 19 de março de 1913, no coração de Paris, foi inaugurada a “nova Pitié”, como “um hospital magnífico, animado pela sua poderosa fábrica, equipado com todos os dispositivos técnicos, ricamente abastecido com laboratórios e salas de operações” (Poincaré, 20 mar. 1913, p.6). Raymond Poincaré, presidente da República francesa, elogiou o trabalho dos construtores realizado ao longo dos anos, sendo descritas a excelência científica e a força de modernização que esse novo hospital representava para as sociedades presentes e futuras. Celebrando a relação com a longa e prestigiosa memória da anterior “velha Pitié”, o discurso estabeleceu um contínuo de solidariedade e dedicação contra o sofrimento e a doença, em consonância com os ideais do regime republicano francês. Os pacientes encontrariam nesse novo complexo hospitalar, em estilo pavilhão, os dispositivos técnicos essenciais para a higiene e o conforto modernos (aquecimento, ventilação, desinfeção, esterilização, distribuição de água quente e casas de banho). A modernidade também estava presente na imagem da “enfermeira moderna”: profissional, secular, inspirada nos modelos anglo-saxónicos, cuidadosa e confortadora dos sofredores. Entre continuidade, reconstrução e transformação, essa inauguração ilustra – apesar dos clichés que transmitiu – a complexidade do local, da organização e da instituição do “hospital” e sua governança. Reuniu diferentes partes interessadas – cartola e fraque – combinando recursos técnicos, financeiros e humanos com ciências médicas, instalações de investigação e de clínica, abertas a várias forças políticas, sociais e ambientais.

Os autores deste suplemento de *História, Ciências, Saúde – Manguinhos* propõem uma abordagem abrangente da complexidade do hospital como objeto histórico, uma reflexão sobre as múltiplas circulações e temporalidades da gestão do hospital, da medicina e da saúde pública na história contemporânea (Granshaw, Porter, 1989). Abordam-se diferentes perspetivas e metodologias variadas no amplo contexto das mudanças científicas, sociais, económicas e políticas ocorridas desde o final do século XIX até ao século XXI (Gorsky, Vilar-Rodríguez, Pons-Pons, 2020). Ao refletir sobre a interação das influências locais e globais, apresenta-se uma narrativa focada nos determinantes da saúde pública. Os artigos aqui reunidos oferecem, assim, uma visão geral da evolução multifacetada da gestão do hospital, na dicotomia local-global (Packard, 2016).

Em “Para uma história da governança hospitalar global, do final do século XIX a meados do século XX: contribuição para uma história a ser escrita”, Céline Paillette introduz a discussão historiográfica sobre os hospitais na história contemporânea. A autora propõe marcos para a história da gestão hospitalar global desde finais do século XIX até meados do século XX, explorando o desenvolvimento histórico e as dimensões internacionais da governança hospitalar. Paillette, além de destacar a interação entre influências locais e globais, redes profissionais e inovação nos cuidados médicos, enfatiza, ainda, a importância de entender a governança hospitalar global como um processo contínuo de ajuste e negociação entre as várias partes interessadas, comparando hospitais a objetos-chave na história da globalização contemporânea, semelhantes a portos e fábricas.

Segue-se um conjunto de artigos que exploram o tema da gestão dos hospitais em Portugal e no Brasil, mostrando de que forma as diferenças regionais nas estruturas de governança influenciaram o desenvolvimento dos sistemas de saúde em momentos e contextos científicos, políticos e sociais específicos.

Alexandra Esteves, em “Os hospitais em Portugal no século XIX e na primeira metade do século XX”, apresenta uma visão geral dos principais hospitais do país e faz uma análise histórica das mudanças políticas e sociais que afetaram o atendimento hospitalar e do envolvimento gradual do Estado na saúde pública. Cada regime trouxe mudanças legislativas, embora nem sempre efetivamente implementadas, para atender às necessidades da sociedade portuguesa. O artigo alude, ainda, aos desafios do setor hospitalar no contexto nacional, designadamente as instalações inadequadas, a falta de recursos e as disparidades sociais no acesso à saúde.

Monique Palma e Isabel Amaral apresentam reflexões sobre o impacto dos avanços científicos, a influência das teorias médicas e o efeito dos fatores sociopolíticos e ambientais na saúde pública, utilizando a história dos principais hospitais do Porto e de Lisboa como estudos de caso. “A influência da teoria miasmática na construção do Hospital Geral de Santo António no Porto, século XIX”, de Monique Palma, reflete sobre a influência da ciência europeia nas práticas médicas locais, destacando a importância dos fatores ambientais na saúde. Palma adota uma abordagem teórica que enfatiza as relações interespecíficas e o papel dos agentes não humanos no desenvolvimento da história humana e das práticas médicas, para destacar influência dos fatores ambientais e dos agentes não humanos na saúde e no *design* do hospital. Em “Hospitais e especialização médica em Lisboa, 1880-1933”, Isabel Amaral procura mostrar como os hospitais de Lisboa influenciaram o desenvolvimento da especialização médica e como esta se tornou a força impulsionadora das reformas dos sistemas de saúde na transição do século XIX para o século XX em Portugal. A separação da Santa Casa da Misericórdia de Lisboa do hospital, em 1851, torna a gestão hospitalar na capital um caso único no sistema hospitalar português. Os desafios enfrentados pelos hospitais da capital (a sobrelotação, a necessidade de cuidados especializados e o impacto das políticas de saúde pública) na administração hospitalar e na assistência médica deram origem à proliferação de pequenos hospitais, do Centro da cidade para as áreas limítrofes, como consequência da especialização médica em consolidação.

Renato da Gama-Rosa Costa, Renata Santos e Giovanna Martire, em “Hospitais de Manguinhos: reflexões sobre políticas científicas e patrimônio, das doenças tropicais à covid-19”, ilustram a influência dos modelos de saúde pública globais nas práticas locais, utilizando como referência dois hospitais no Rio de Janeiro: o atual Hospital Evandro Chagas e o Centro Hospitalar Covid-19, ambos construídos para dar resposta a crises sanitárias emergentes. O Hospital Evandro Chagas, construído em 1918, destinava-se ao tratamento de doenças tropicais, nomeadamente a doença de Chagas, e refletia os avanços médicos do início do século XX. O segundo hospital foi construído em 2020, em apenas 60 dias, como resposta à pandemia da covid-19. Os autores discutem a importância da valorização desses dois espaços icónicos da saúde pública no Brasil, a partir de considerações científicas, arquitetónicas, sanitárias, patrimoniais e políticas.

“O ‘pharmakon’ da prevenção: imunidade artificial na pandemia de covid-19”, de Sofia Varino, oferece uma análise abrangente das medidas de prevenção da covid-19 e discute suas implicações e os contextos sociopolíticos e culturais em que foram aplicados. Utilizando o conceito de *pharmakon*, que encapsula a ambivalência paradoxal das intervenções terapêuticas tendo efeitos tanto prejudiciais quanto benéficos, Varino estabelece um paralelo das narrativas e práticas em torno do uso de Salvarsan para sífilis e do desenvolvimento das vacinas anticovid-19. O artigo realça a necessidade de uma abordagem mais subtil e equitativa para a saúde pública, considerando os contextos sociopolíticos e económicos das intervenções biomédicas, ao mesmo tempo que destaca a integração de perspetivas ambientais e ecológicas nos modelos de saúde pública, como a abordagem de One Health.

É também analisado um conjunto de fotografias de autoria de um fotógrafo médico amador que ilustram o estado da saúde e dos hospitais em Lisboa no início do século XX. Manuel Mendes Silva, em “A saúde e a medicina portuguesas no primeiro quartel do século X pelas lentes de Jorge Marçal da Silva”, apresenta uma narrativa visual que mostra as condições dos hospitais, o impacto dos avanços médicos, as realidades cotidianas e o olhar médico em Portugal. As fotografias são imagens reveladoras das realidades da vida hospitalar e dos desafios colocados à saúde pública em Portugal, que complementam o entendimento das práticas médicas e do papel dos hospitais em Lisboa nas primeiras décadas do século XX.

Embora este suplemento apresente uma visão abrangente do desenvolvimento histórico da gestão hospitalar, outras áreas carecem de investigação, como o impacto da globalização na gestão hospitalar, o papel da defesa e do empoderamento dos pacientes, bem como a influência das mudanças sociopolíticas no desenvolvimento da rede de assistência hospitalar. Reconhecer os pacientes como parte-chave interessada no sistema de saúde tem implicações significativas para a gestão dos hospitais, e entender como incorporar as vozes desses atores nas estruturas de gestão é crucial para dar resposta aos desafios atuais e futuros da assistência médica pública.

Explorando a complexa interação entre influências locais e globais, mudanças culturais e sócio-políticas e progressos no conhecimento científico e tecnológico, em suma, os múltiplos significados da gestão da saúde global, esperamos que as reflexões históricas dos artigos aqui reunidos contribuam para uma discussão abrangente dos desafios da saúde pública no presente e no futuro.

Agradecemos a todos os autores pelas suas contribuições e aos pareceristas pelos seus comentários, que muito colaboraram para melhorar este trabalho. Esperamos que este suplemento de *História, Ciências, Saúde – Manguinhos* motive outros trabalhos e novas perspetivas de investigação que enriqueçam o debate sobre os temas aqui tratados.
