# Supplementary material for: A EVOLUÇÃO DO HOSPITAL E A GESTÃO DA SAÚDE: ENTRE O LOCAL E O GLOBAL
Source: Hist Cienc Saude Manguinhos. 2024 Oct 14;31(Suppl 1):e2024050. [Article in Portuguese] doi: 10.1590/S0104-59702024000100050 (PMC11472720; doi:10.1590/S0104-59702024000100050)
Supplement: Translation

# Translation: THE EVOLUTION OF HOSPITAL AND HEALTH GOVERNANCE: BETWEEN LOCAL AND GLOBAL

**DOI:** 10.1590/S0104-59702024000100050en

**Authors:** Isabel Amaral, Alexandra Esteves, Céline Paillette

**Affiliations:** i Guest editor, Centro Interuniversitário de História das Ciências e da Tecnologia/ NOVA Faculdade de Ciências e Tecnologia. Lisboa – Portugal; ii Guest editor, Universidade do Minho. Braga – Portugal; iii Guest editor, Université Paris 1-Panthéon Sorbonne. Paris – France

On March 19, 1913, in the heart of Paris, the “new Pitié” was inaugurated as “a magnificent hospital, animated by its powerful machinery, equipped with all technical devices, and richly furnished with laboratories and operating rooms” (Poincaré, 20 mar. 1913, p.6). Raymond Poincaré, president of the French Republic, praised the builders’ work over the years, describing the scientific excellence and modernization that this new hospital represented for both present and future societies. Celebrating the relationship with the long-standing and prestigious memory of the former “old Pitié,” this speech established a continuum of solidarity and dedication against suffering and disease, in line with the ideals of the French republican regime. The patients would find in this new pavilion-style hospital complex the technical devices essencial for modern hygiene and comfort (heating, ventilation, disinfection, sterilization, hot water distribution, and bathrooms). Modernity was also reflected in the image of the “modern nurse:” professional, secular, inspired by Anglo-Saxon models, caring, and comforting to those who suffered. Between continuity, reconstruction, and transformation, this inauguration illustrated – despite the clichés it conveyed – the complexity of the place, the organization, and the institution of the “hospital” and its governance. It brought together different stakeholders – top hat and white coat – in combining technical, financial, and human resources with medical sciences, research and clinical facilities, open to various political, social, and environmental forces.

The authors of this supplement of *História, Ciências, Saúde – Manguinhos* propose a comprehensive approach to the complexity of the hospital as a historical object, reflecting on the multiple circulations and temporalities of hospital management, medicine, and public health in contemporary history (Granshaw, Porter, 1989). Different theoretical perspectives methodologies are used within the broader context of scientific, social, economic, and political changes from the late nineteenth century to the twenty-first (Gorsky, Vilar-Rodríguez, Pons-Pons, 2020). By reflecting on the interaction of local and global influences, a narrative focused on the determinants of public health is presented. These articles thus offer an overview of the multifaceted evolution of hospital management in the local-global dichotomy (Packard, 2016).

In “Towards a history of global hospital governance, late nineteenth century to mid-twentieth century: a contribution to a history yet to be written,” Céline Paillette introduces the historiographical discussion on hospitals in contemporary history. The author proposes frameworks for the history of global hospital management from the late nineteenth century to the mid-twentieth century, exploring the historical development and international dimensions of hospital governance. Paillette, in addition to highlighting the interaction between local and global influences, professional networks, and innovation in medical care, also emphasizes the importance of understanding global hospital governance as a continuous process of adjustment and negotiation among various stakeholders. She compares hospitals to key objects in the history of contemporary globalization, such as ports and factories.

The following articles explore the topic of hospital management in Portugal and Brazil, demonstrating how regional differences in governance structures influenced the development of health systems in specific scientific, political, and social contexts and periods.

In “Hospitals in Portugal from the nineteenth to the mid-twentieth centuries”, Alexandra Esteves presents an overview of the country’s main hospitals and provides a historical analysis of the political and social changes that affected hospital care and the gradual involvement of the State in public health. Each regime brought legislative changes, though not always effectively implemented, to meet the needs of Portuguese society. The article also alludes to the challenges of the hospital sector in the national context, namely inadequate facilities, lack of resources, and social disparities in access to healthcare.

Monique Palma and Isabel Amaral present reflections on the impact of scientific advances, the influence of medical theories, and the effect of sociopolitical and environmental factors on public health, using the history of the main hospitals in Porto and Lisbon as case studies. “The influence of miasmatic theory on the construction of the Hospital Geral de Santo António in Porto in the nineteenth century,” by Monique Palma, discusses the influence of European science on local medical practices, highlighting the importance of environmental factors in health. Palma adopts a theoretical approach that emphasizes the interspecific relationships and the role of non-human agents in the development of human history and medical practices to draw attention to the influence of environmental factors and non-human agents on health and hospital design. In “Hospitals and medical specialization in Lisbon, 1880-1933,” Isabel Amaral seeks to show how hospitals in Lisbon influenced the development of medical specialization and how it became the driving force behind health system reforms in the transition from the nineteenth to the twentieth century in Portugal. The separation of the Santa Casa da Misericórdia of Lisbon from the hospital, in 1851, made hospital management in the capital a unique case in the Portuguese hospital system. The challenges faced by the capital’s hospitals (overcrowding, the need for specialized care, and the impact of public health policies) in hospital administration and medical care led to the proliferation of small hospitals, from the city center to the surrounding areas, as a consequence of consolidating medical specialization.

Renato da Gama-Rosa Costa, Renata Santos and Giovanna Martire, in “Manguinhos hospitals: reflections on scientific politics and heritage, from tropical diseases to covid-19,” illustrate the influence of global public health models on local practices, using two hospitals built in Rio de Janeiro as reference: the present Evandro Chagas Hospital, and the Covid-19 Hospital Center, both built to respond to emerging health crises. The Evandro Chagas Hospital, built in 1918, was intended for the treatment of tropical diseases, particularly Chagas disease, and reflected the medical advancements of the early twentieth century. The second hospital was built in 2020, in just 60 days, in response to the covid-19 pandemic. The authors discuss the importance of valuing these two iconic public health spaces in Brazil, from scientific, architectural, sanitary, heritage, and political perspectives.

“The ‘pharmakon’ of prevention: artificial immunity in the covid-19 pandemic,” by Sofia Varino, offers a comprehensive analysis of covid-19 prevention measures, and discusses their implications and the socio-political and cultural contexts in which they were applied. Using the concept of *pharmakon*, which encapsulates the paradoxical ambivalence of therapeutic interventions having both harmful and beneficial effects, Varino draws parallels between the narratives and practices surrounding the use of Salvarsan for syphilis and the development of covid-19 vaccines. The article highlights the need for a more nuanced and equitable approach to public health, considering the socio-political and economic contexts of biomedical interventions, while stressing the integration of environmental and ecological perspectives into public health models, such as the One Health approach.

A set of photographs by an amateur medical photographer illustrating the state of health and hospitals in Lisbon during the twentieth century is also analyzed. Manuel Mendes Silva, in “Portuguese health and medicine during the first quarter of the twentieth century through the lenses of Jorge Marçal da Silva,” presents a visual narrative that shows the conditions of hospitals, the impact of medical advances, the everyday realities and the medical gaze in Portugal. The photographs are revealing images of the realities of hospital life and the challenges posed to public health in Portugal, which complement the understanding of medical practices and the role of hospitals in the first decades of the twentieth century.

While this special issue provides a comprehensive overview of the historical development of hospital management, other areas need further research, such as the impact of globalization on hospital management, the role of patient advocacy and empowerment, and the influence of socio-political changes on the development of the hospital care network. Recognizing patients as a key stakeholder in the healthcare system has significant implications for hospital management, and understanding how to incorporate the voices of these actors into management structures is crucial to meeting the current and future challenges of public healthcare.

By exploring the complex interaction between local and global influences, cultural-socio-political changes and advances in scientific and technological knowledge, in short, the multiple meanings of global health management, we hope that the historical reflections of the articles presented here will contribute to a comprehensive discussion of the challenges of public health in the present and future.

We would like to thank all the authors for their contributions and the peer-reviewers for their comments, which have greatly improved this work. We hope that this special issue of *História, Ciências, Saúde – Manguinhos* motivate other works and new research perspectives that will enrich the debate on the topics covered here.

## References

[B11] GORSKY Martin, VILAR-RODRÍGUEZ Margarita, PONS-PONS Jerònia (2020). The political economy of the hospital in history.

[B22] GRANSHAW Lindsay, PORTER Roy (1989). The hospital in history.

[B33] PACKARD Randall (2016). A history of global health: interventions into the lives of other people.

[B44] POINCARÉ Raymond (1913). Inauguration de la Nouvelle Pitié. Le Temps.
